# Diacylglycerol-Stimulated Endocytosis of Transferrin in Trypanosomatids Is Dependent on Tyrosine Kinase Activity

**DOI:** 10.1371/journal.pone.0008538

**Published:** 2010-01-01

**Authors:** Sandesh Subramanya, Kojo Mensa-Wilmot

**Affiliations:** Department of Cellular Biology, University of Georgia, Athens, Georgia, United States of America; Research Institute for Children and the Louisiana State University Health Sciences Center, United States of America

## Abstract

Small molecule regulation of cell function is an understudied area of trypanosomatid biology. In *Trypanosoma brucei* diacylglycerol (DAG) stimulates endocytosis of transferrin (Tf). However, it is not known whether other trypanosomatidae respond similarly to the lipid. Further, the biochemical pathways involved in DAG signaling to the endocytic system in *T. brucei* are unknown, as the parasite genome does not encode canonical DAG receptors (*e.g.* C1-domains). We established that DAG stimulates endocytosis of Tf in *Leishmania major*, and we evaluated possible effector enzymes in the pathway with multiple approaches. First, a heterologously expressed glycosylphosphatidylinositol phospholipase C (*GPI-PLC*) activated endocytosis of Tf 300% in *L. major*. Second, exogenous phorbol ester and DAGs promoted Tf endocytosis in *L. major*. In search of possible effectors of DAG signaling, we discovered a novel C1-like domain (*i.e.* C1_5) in trypanosomatids, and we identified protein Tyr kinases (PTKs) linked with C1_5 domains in *T. brucei*, *T. cruzi*, and *L. major*. Consequently, we hypothesized that trypanosome PTKs might be effector enzymes for DAG signaling. General uptake of Tf was reduced by inhibitors of either Ser/Thr or Tyr kinases. However, DAG-stimulated endocytosis of Tf was blocked only by an inhibitor of PTKs, in both *T. brucei* and *L. major*. We conclude that (i) DAG activates Tf endocytosis in *L. major*, and that (ii) PTKs are effectors of DAG-stimulated endocytosis of Tf in trypanosomatids. DAG-stimulated endocytosis of Tf may be a *T. brucei* adaptation to compete effectively with host cells for vertebrate Tf in blood, since DAG does not enhance endocytosis of Tf in human cells.

## Introduction

Endocytosis in eukaryotes is important for uptake of nutrients (*e.g*. iron, and cholesterol esters), maintenance of cell volume, and for modulation of cell signaling (reviewed in [1]). Lipids regulate various steps of endocytosis.

Diacylglycerol (DAG) is a second messenger for cell signaling. Receptors and effectors, the best known of which is protein kinase C (PKC), mediate signaling by DAG. C1-domain proteins bind DAG (and phorbol ester) [2]. Non-kinase receptors of DAG include chimaerins, CalDAG-GEF1, and RasGRP.

Glycosylphosphatidylinositol phospholipase C (GPI-PLC) is expressed in bloodstream *T. brucei*. Products of the enzyme's digestion of GPI include diacylglycerols (DAGs) and inositolphosphoglycans [3], [4], [5], [6]. GPI-PLC can cleave intracellular GPIs at the endoplasmic reticulum [6], and regulates endocytosis of transferrin (Tf), the iron-binding protein, in bloodstream *T. brucei* [7]. In a mouse model of human African trypanosomiasis, the enzyme contributes to virulence of a pleomorphic strain of *T. brucei* [8].

DAG (or phorbol ester) stimulates endocytosis of Tf in *T. brucei* [7]. The enzyme is not important for release of GPI-anchored variant surface glycoprotein (VSG) from the plasma membrane [8], [9]. However, DAG regulation of Tf endocytosis in other trypanosomatids has not been evaluated. Further, the signaling pathway used by DAG to stimulate endocytosis in *T. brucei* is not known.

*Leishmania* are trypanosomatid protozoans that acquire host hemoglobin and transferrin by endocytosis [10], [11], [12], [13], [14]. Currently, no lipid regulators of endocytosis have been described in *Leishmania*. In this work, we used *L. major* as a model trypanosomatid to study DAG-stimulated Tf endocytosis. Heterologous (stable) expression of a *GPI-PLC* gene in *L. major* promoted endocytosis of Tf. In addition, preincubation of *L. major* with DAG or phorbol ester increased endocytosis of Tf. These data document DAG regulation of Tf endocytosis in *L. major*, and establish DAG as a signaling lipid in the parasite.

To gain insight into signaling pathways used by DAG to activate Tf endocytosis in trypanosomatids we used a combination of bioinformatic and pharmacological approaches. We discovered a novel C1-like domain linked to protein Tyr kinases in *T. brucei*: And, in both *L. major* and *T. brucei* an inhibitor of protein Tyr kinase (PTK) arrested DAG-stimulated endocytosis of Tf. These data indicate that DAG signaling to the endocytic system in trypanosomatids is regulated by PTKs. Consistent with this model, the genomes of *T. brucei* and *L. major* do not encode Ser/Thr kinases with C1-domains (i.e., protein kinases C) that could be effectors of the actions of DAG in these deeply-diverged eukaryotes.

## Results

### Transferrin Endocytosis in *L. major* Is Stimulated by a GPI-Phospholipase C

*Leishmania* acquire host transferrin (and indirectly iron) by endocytosis [15], [16]. A 70 kD Tf-binding protein has been implicated in its uptake but mechanisms regulating acquisition of Tf by *Leishmania* spp have not been studied.

**Figure 1:**
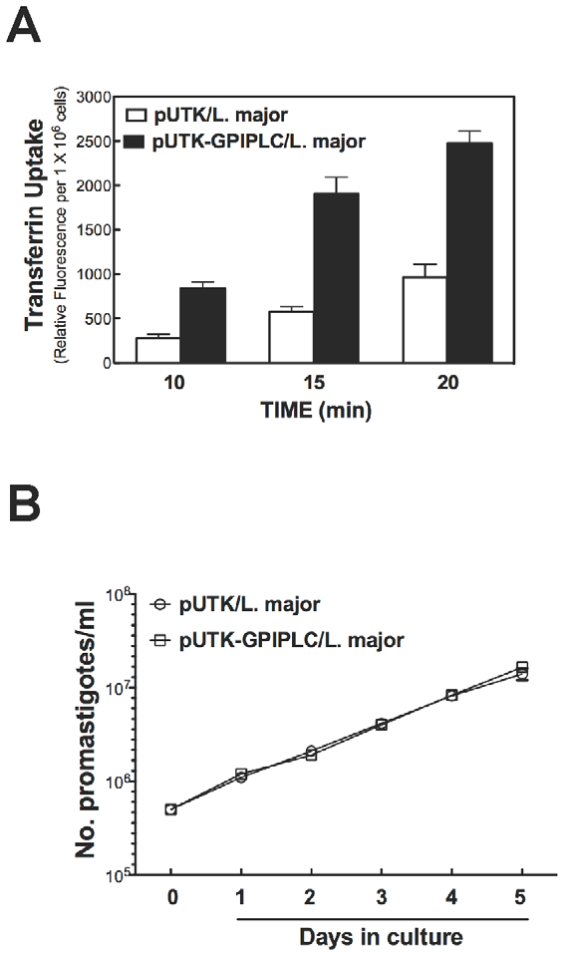
Heterologously expressed GPI-PLCp activates endocytosis of Tf in *Leishmania major*. *(* ***A*** *) Leishmania major* CC1 promastigotes expressing GPI-PLC (pUTK-GPIPLC) or vector alone (pUTK) were cultured in a medium containing G418 (50 µg/ml). Cells (1×10^6^/ml) were incubated with transferrin-Alexa Fluor 594 (25 µg/ml) at 27°C, and at specified time intervals cell-associated fluorescence was measured. Relative Fluorescence Units was plotted after subtracting background fluorescence of an equivalent number of control cells. Mean±standard deviation of triplicate determinations are plotted. *(* ***B*** *)* Promastigote *L. major* (pUTK/*L. major* and pUTK-GPIPLC/*L. major*) were seeded at a density of 5×10^5^ cells/ml in medium containing G418 (50 µg/ml), and every 24 h cells were counted microscopically. Mean±standard deviation of duplicate measurements is plotted.

GPI-phospholipase C (GPI-PLC) from *T. brucei* activates endocytosis of transferrin in that parasite [7] most likely by releasing DAG from cleavage of GPIs in the trypanosome. We tested a possibility that endocytosis of Tf in *L. major* would be affected by presence of GPI-GPI-PLC polypeptide (GPI-PLCp). (The *L. major* genome does not encode a *GPI-PLC* gene.) For this objective, *L. major* stably harboring plasmid pUTK-GPIPLC (pUTK-GPIPLC/*L.major*) and a control strain transfected with the vector alone (pUTK/*L.major*) were used in Tf endocytosis assays. *L. major* expressing GPI-PLC accumulated 200–300% more transferrin-Alexa Fluor 594 than control pUTK/*L. major* (Fig. 1A). Hence a GPI-PLC can regulate Tf endocytosis in *L. major*. GPI-PLC expression had no effect on the growth rate of *L. major* (Fig. 1B).

When expressed in *L. major*, GPI-PLCp can be directed either to endosomes or to glycosomes (peroxisomes). The unmutated enzyme associates with endosomes, whereas Cys-to-Ser mutations at positions 269, 270, and 273 in GPI-PLCp targets the protein to endosomes [17]. We tested whether different sub-cellular locations of enzymatically active Cys mutants of GPI-PLCp affected Tf endocytosis in *L. major*.

**Figure 2:**
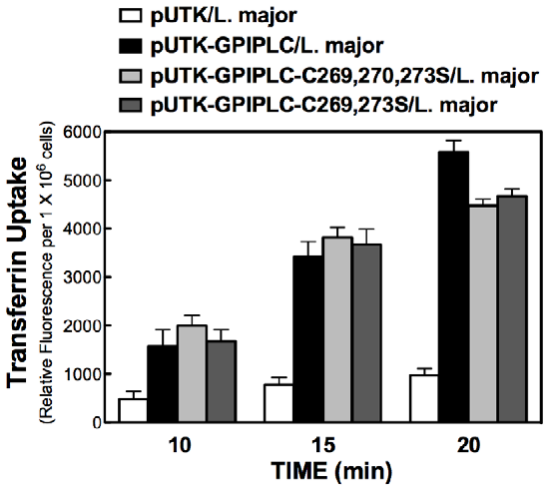
Endosome or glycosome-directed GPI-PLC stimulate endocytosis. *Leishmania* harboring pUTK-GPIPLCp, pUTK-GPIPLC-C269S/C273S, pUTK-GPIPLC-C269S/C270S/C273S, or pUTK-GPIPLC-Q81L were cultured in medium containing 50 µg/ml G418 and transferrin endocytosis was measured as described in Fig. 1. Representative data from three experiments is shown. (Intracellular location of GPI-PLCp and its Cys mutants has been described [6], [43].)

Unmutated GPI-PLCp facilitated Tf uptake in *L. major* (Fig. 1). Similarly, in pUTK-GPIPLC-C269,273S/*L.major* and pUTK-GPIPLC-C269,270,273S/*L.major* uptake of Tf was increased in comparison to *L. major* expressing vector (pUTK/*L.major*) alone (Fig. 2). Tf accumulation in these Cys mutants was comparable to levels observed with *L. major* expressing unmutated GPI-PLC. We conclude that glycosome location of GPI-PLCp is equally effective as endosomal GPI-PLCp in stimulating uptake of Tf into *L. major*. Thus endosome localization of GPI-PLCp is not required for the enzyme's stimulation of Tf endocytosis in *L. major*.

### Enzyme Activity Is Important for GPI-PLC Stimulation of Tf Endocytosis in *L. major*

Since GPI-PLC promoted Tf endocytosis from either endosomes or glycosomes, we considered a hypothesis that the enzyme produced a diffusible second messenger (e.g. DAG) that mediated its physiological effect. As a first step to evaluate this theory, we analyzed the effect of an enzymatically inactive Gln81Leu (Q81L) GPI-PLCp mutant [18] on Tf endocytosis in *L. major*.

**Figure 3:**
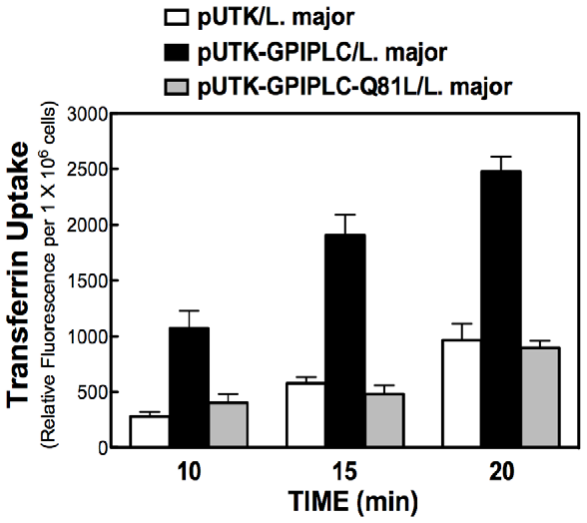
Enzyme activity is important for GPI-PLCp stimulation of Tf Endocytosis in *L. major*. *L. major* pUTK/GPIPLC-Q81L and pUTK-GPIPLC were cultured in 50 µg/ml G418 and allowed to endocytose transferrin-Alexa Fluor 594 at 27°C for indicated time intervals. Cell-associated transferrin is plotted as relative fluorescence units.

The enzymatically inactive GPI-PLCp mutant (i.e. pUTK-Q81L_GPIPLC) did not augment Tf uptake in *L. major*, as the amount of Tf endocytosis in pUTK-Q81L_GPIPLC/*Lmajor* was comparable to that obtained from cells expressing the vector (pUTK) alone (Fig. 3). These data suggest that a product of GPI-PLC enzyme activity (*i.e.* either DAG or inositolphosphoglycan) is probably needed for the enzyme's effect on Tf endocytosis.

### Phorbol Ester or Diacylglycerols Stimulate Endocytosis of Tf in *L. major*

Since enzyme activity is important for GPI-PLCp activation of endocytosis we tested if a lipid product of GPI cleavage (*i.e.* DAG) was a second messenger for the enzyme. Phorbol esters bind C1-domains and are used to characterize DAG signaling pathways [19]. Therefore we tested whether phorbol ester or DAG would stimulate endocytosis of Tf in *L. major*.

**Figure 4:**
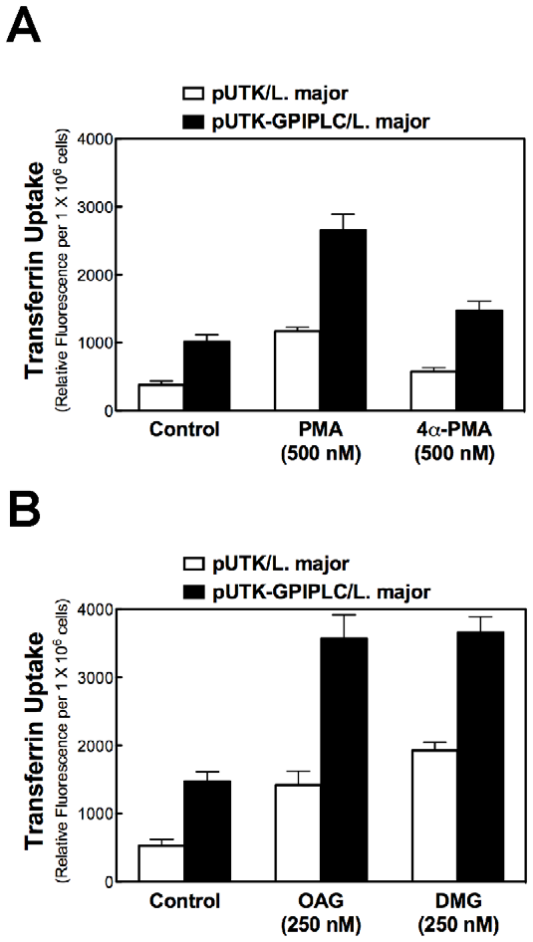
Phorbol ester and diacylglycerols activate endocytosis of Tf in *L. major*. (***A***) *L major* harboring *pUTK* or *pUTK-GPIPLC* (1×10^6^/ml) were incubated at 27°C with PMA or 4α-PMA (500 nM; final concentration) for 15 min, or (***B***) oleoyl-acetyl-sn-glycerol (OAG) or dimyristoyl glycerol (DMG) (250 nM; final concentration) for 30 min. Endocytosis of Tf was measured as described in the legend for Figure 1. Data presented are means (with standard deviations) of triplicate determinations.

Pre-incubation of *L. major* harboring either pUTK or pUTK-GPIPLC with phorbol-12-myristate-13-acetate (PMA) increased Tf endocytosis two to three-fold (Fig. 4A) in comparison to control cells that were treated with vehicle (DMSO). In another control experiment, the alpha isomer of PMA (i.e. 4α-PMA) did not stimulate endocytosis of Tf (Fig. 4A), indicating that the effects of PMA are limited to the physiologically active 4β-PMA isomer [20].

The results from the PMA studies were confirmed with experiments involving DAGs. For this objective, either OAG (1-oleoyl-2-acetyl-sn-glycerol) or DMG (1,2-dimyristoyl-sn-glycerol) was pre-incubated with *L. major* for 30 min prior to endocytosis of Tf. OAG or DMG (250 nM) stimulated Tf endocytosis three-fold in *L. major* (as compared to untreated cells (Fig. 4B)). These data confirm that a (i) DAG regulates Tf endocytosis in *L. major*, and (ii) DAG is a second messenger in *L. major*.

### Protein Kinase C (PKC) Is Not Required for PMA-Stimulated Endocytosis

**Figure 5:**
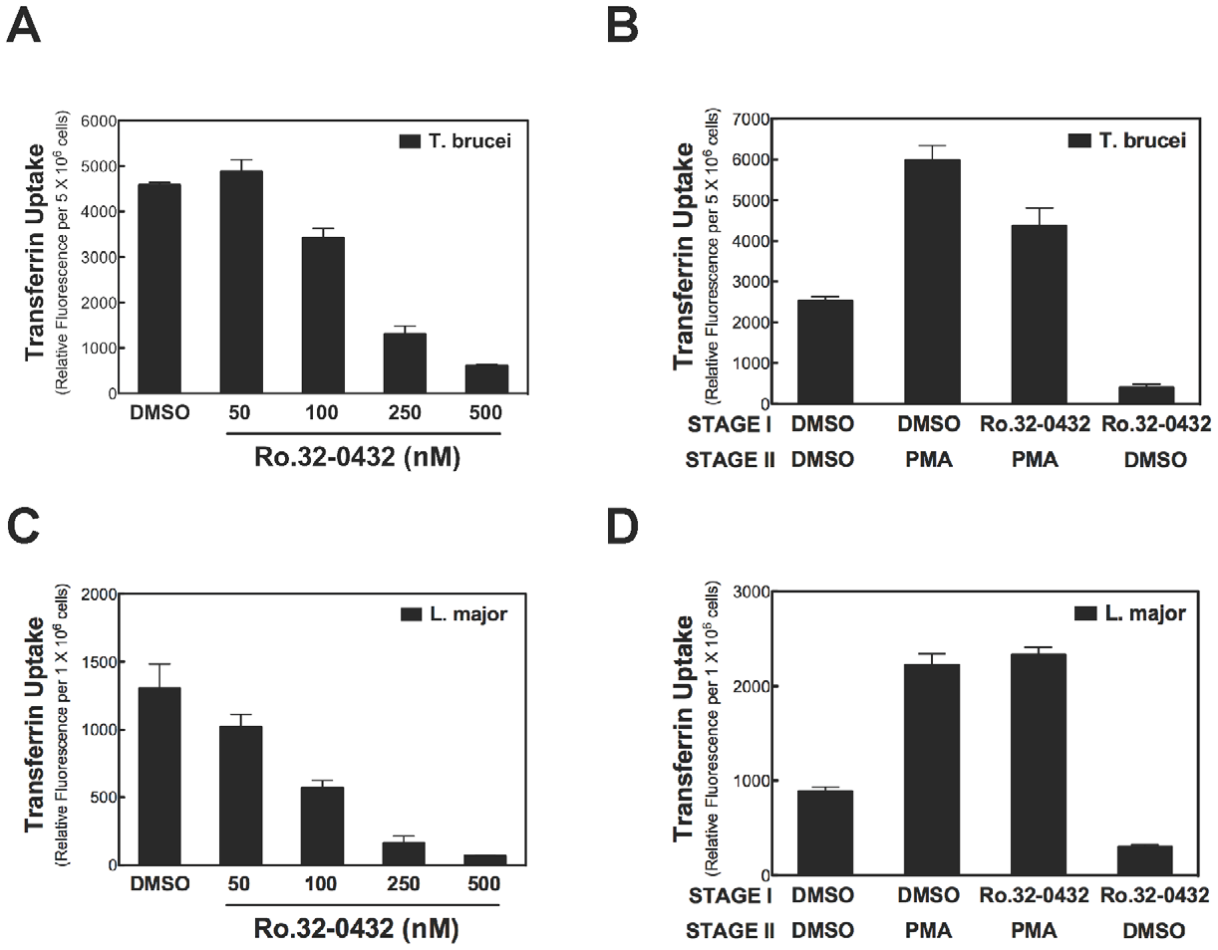
A Ser/Thr kinase Inhibitor does not block DAG-activated endocytosis of Tf in *T. brucei* or *L. major*. Bloodstream *T. brucei* (5×10^6^ cells) *(* ***A*** *)* or *L. major* promastigotes (1×10^6^ cells) *(* ***C*** *)* were incubated with DMSO (vehicle) or different amounts of Ro32-0432 for 10 min at 37°C (for *T. brucei*) or 27°C (for *L. major*). Subsequently, endocytosis of Tf was measured as described earlier. *T. brucei (* ***B*** *)* or *L. major (* ***D*** *)* was incubated in medium containing Ro32-0432 (500 nM) for 10 min (i.e., Stage I). Cells were then exposed to PMA (500 nM) (Stage II) for another 10 min, and endocytosis of Tf was measured. A representative experiment is presented. Data plotted are means (with standard deviations) of triplicate determinations.

PKC is a Ser/Thr kinase that mediates many physiological effects of DAG in vertebrates [21]. Therefore, we tested whether DAG-activated endocytosis in *Leishmania* or *T. brucei* required a PKC. Ro32-0432, a cell-permeable active site-directed PKC inhibitor [22], [23] was used for our study. We first tested whether general uptake of Tf in *T. brucei* was inhibited by Ro32-0432. Towards this goal, cells were pre-incubated with the compound and endocytosis of Tf was measured afterwards. Ro32-0432 inhibited Tf endocytosis in *T. brucei* with an IC_50_ (concentration of the inhibitor needed to reduce Tf endocytosis by 50%) of 200 nM (Fig. 5A). This result indicates that a Ser/Thr kinase is involved in some aspect of the uptake Tf in the trypanosome.

We next investigated whether PMA-stimulated endocytosis of Tf in *T. brucei* depended on a Ser/Thr kinase. A C1-domain of PKC binds PMA (reviewed in [24], [25]). Therefore, involvement of PKC in cellular events is obtained by sequential administration of an active-site directed inhibitor of PKC (*e.g.* Ro32-0432) and a phorbol ester [26], [27]. (Ro32-0432 will inhibit a Ser/Thr kinase whose active site architecture is similar to that of PKC even if the enzyme lacks a C1-domain). For our purposes, *T. brucei* was incubated with Ro32-0432 in a first stage. Afterwards, PMA was added to the cells in the second stage, and then endocytosis of Tf was measured at the final stage (Fig. 5B).

Added alone to *T. brucei*, Ro32-0432 inhibited whereas PMA activated endocytosis of Tf (Fig. 5B). When *T. brucei* was pre-treated with Ro32-0432, PMA activated Tf endocytosis 500%, as compared to cells that were not treated with PMA after addition of the kinase inhibitor (Fig. 5B). We conclude that Ro32-0432 cannot block PMA activation of Tf endocytosis. Therefore, the pathway for PMA-regulated endocytosis of Tf does not involve a PKC in *T. brucei*. However, a Ser/Thr kinase is important in a general pathway for endocytosis of Tf that is not dependent on DAG (Fig. 5A).

In *L. major*, similar results were obtained when the effect of Ro32-0432 on PMA-activated endocytosis of Tf were studied. Ro32-0432 inhibited Tf endocytosis (IC_50_ = 100 nM) (Fig. 5C). Strikingly, PMA still activated Tf endocytosis even when added after *L. major* had been preincubated Ro32-0432 (Fig. 5D). Thus, PMA stimulation of Tf endocytosis in *L. major* does not depend a PKC-like Ser/Thr kinase.

### Identification of a Novel C1-Like Domain in Trypanosomatids

Having demonstrated that DAG regulates endocytosis of Tf in *T. brucei*, we expected to find C1-domains that are used in vertebrates for recognition of the lipid encoded in the genome of the parasite. Surprisingly, no C1-domains are annotated in *T. brucei* [28]. Thus, the bioinformatics predictions appear to be at odds with our experimental data. To resolve this conundrum, we hypothesized that C1-domains in trypanosomes may not have been discovered because they have diverged significantly from the vertebrate C1-domain to which they were compared during genome annotation. That is, the protein family (Pfam) “scores” of trypanosome C1-domains failed to meet the “gathering cut-off” required for their annotation [29]. To test this theory we, in collaboration with Gaelle Blandin (The Institute of Genomic Research), implemented a genome-wide search for C1-like domains at low stringency. That effort produced 21 hits (not presented) of which those with E-values<0.02 [30] were analyzed further with Pfam [29]. Top scoring trypanosome proteins from that analysis are presented in Table 1.

**Table 1:** C1_5 (C1-like) Domains in *T. brucei*.

Systematic ID	Residues, C1-like	Other Pfam Domains (residues)
Tb11.01.2290	595–628	Pkinase_Tyr (86–235)
Tb03.30P12.1230	795–825	Helicase_C (557–636)
Tb09.211.4210	989–1016	HECT (1442–1912)
Tb08.4A8.390	49–77	None
Tb.10.6k15.2660	23–55	None
Tb10.6k15.0350	185–218	PHD (186–236)
Tb08.2909.320	3550–3590	HECT (3999–4304)
Tb05.26K5.230	193–222	SWIM
Tb08.10K10.560	966–1006	Clathrin_repeat

***Protein Domains:***

CLATHRIN REPEAT (PF00673): Occur in the arm region of the Clathrin heavy chain and VPS (vacuolar protein sorting) proteins.

HECT (PF00632): HECT-domain. Found in ubiquitin-protein E3 ligase that transfers ubiquitin to substrates.

HELICASE_C (PF00271): The signature pattern for this family is [LIVMF]-x-(2)-D-E-A-D-[RKEN]-x-[LIVMFYGSTN]). It is found in a wide variety of helicase related proteins.

SWIM: SWIM is Zn-chelating domain found in a variety of prokaryotic and eukaryotic proteins.

PKINASE_TYR (PF07714): Protein Tyrosine Kinase.

**Figure 6:**
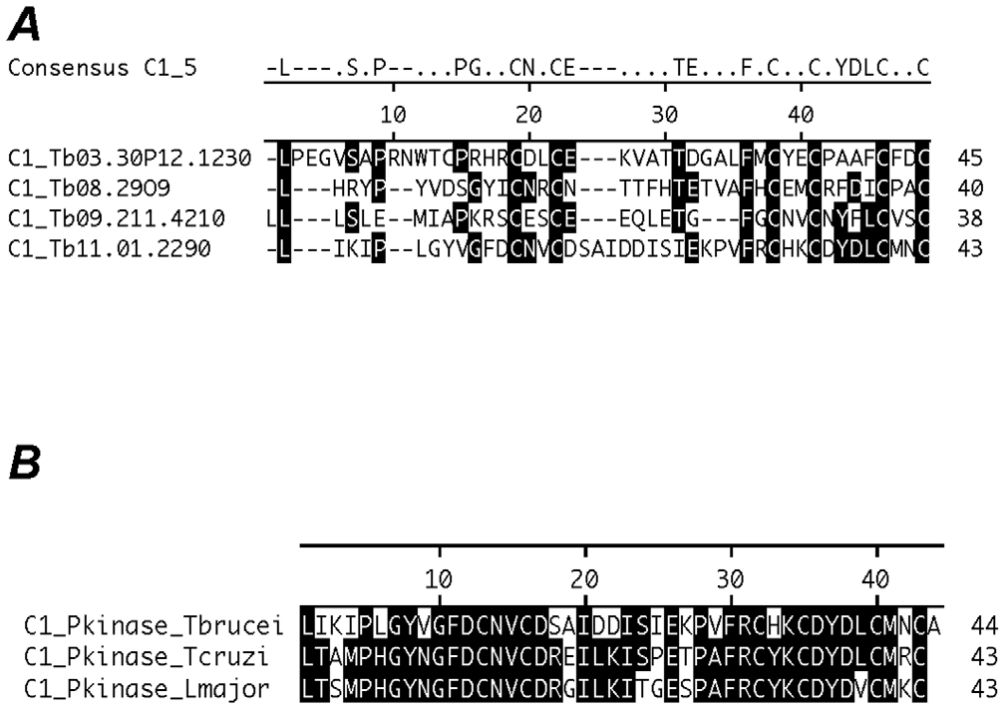
Alignment of C1_5 domains in *T. brucei*. *(* ***A*** *)* C1-like domains of *T. brucei* proteins (Table 1) are aligned (Clustal W) with LaserGene (DNAStar). A consensus sequence is presented above the alignment. *(* ***B*** *)* Alignment of C1_5 domains in protein Tyr kinases from *T. brucei*, *T. cruzi* and *L. major* (see text for details).

We hypothesized that C1-like domains in *T. brucei* (Table 1) might contain a unique peptide motif. To examine this theory, we tested whether the four highest scoring C1-like domains from *T. brucei* (Table 1) contained a recognizable pattern of amino acid residues, by extracting amino acid patterns in the *T. brucei* C1-like domain with the algorithm PRATT [31]. The *T. brucei* C1-like domains have six conserved cysteines (in bold) as part of the pattern L-x(9,12)-**C**-x(2,4)-**C**-x(3,9)-E-x(2,9)-F-x-**C**-x(2)-**C**-x(4)-**C**-x(2)-**C** (PROSITE nomenclature [32], [33]). An alignment of the *T. brucei* C1-like domains is presented in Fig. 6A. Compared to a human C1-domain, H-x-[LIVMFYW]-x(8,11)-**C**-x(2)-**C**-x(3)-[LIVMFC]-x(5,10)-**C**-x(2)-**C**-x(4)-[HD]-x(2)-**C**-x(5,9)-**C**, the *T. brucei* motif lacks a His residue at the N-terminus. In addition, the spacing between the six Cys residues is different in the two domains. For these reasons, we surmise that the *T. brucei* C1-like domain that we term C1_5 (Fig. 6A) has diverged significantly from the (classic) vertebrate C1-domain.

We extended our studies by checking whether C1_5 domains were present in other trypanosomatids. Motif searches of the genomes of *L. major* and *T. cruzi* revealed that each organism contained a protein tyrosine (Tyr) kinase (PTK) with a C1_5 domain, namely LmjF36.5350 in *L. major*, and Tc00.1047053510285.70 in *T. cruzi*. An alignment of the C1_5 domains from PTKs of the trypanosomatids is presented in Fig. 6B.

### Inhibition of Protein Tyrosine Kinase Blocks PMA-Stimulated Tf Endocytosis

We speculated that effector proteins in *T. brucei* might use C1_5 domains that binds DAG to activate proteins in the endocytic system for uptake of Tf. First, we postulated that C1_5 proteins could interact directly with major polypeptides that are required for endocytosis, and influence activity of those proteins. Second, we envisioned that a C1_5 protein might have a second domain with enzyme activity that might be activated by DAG to post-translationally modify proteins involved in endocytosis thereby enhancing Tf uptake. Our second hypothesis is based on a discovery that three C1_5 proteins have domains with enzyme activity, namely, a PTK Tb11.01.2290, and the ubiquitin ligases Tb09.211.4210 and Tb08.2909.320 (Table 1). In vertebrates, Tyr phosphorylation of some receptors modulates their endocytosis. And, ubiquitination of membrane proteins in yeasts and vertebrates accelerates their recruitment into endosomes. Therefore, we hypothesized that DAG might modulate endocytosis in *T. brucei* by influencing activity of PTKs. We used a pharmacological tools to evaluate (i) whether a *T. brucei* PTK (TbPTK) affects Tf endocytosis, and (ii) whether PMA-activated endocytosis of Tf requires TbPTK activity. Tyrphostin A47 (TphA47) an inhibitor of PTKs in vertebrate cells [34] and in *T. brucei* [35], [36] was used for these studies.

**Figure 7:**
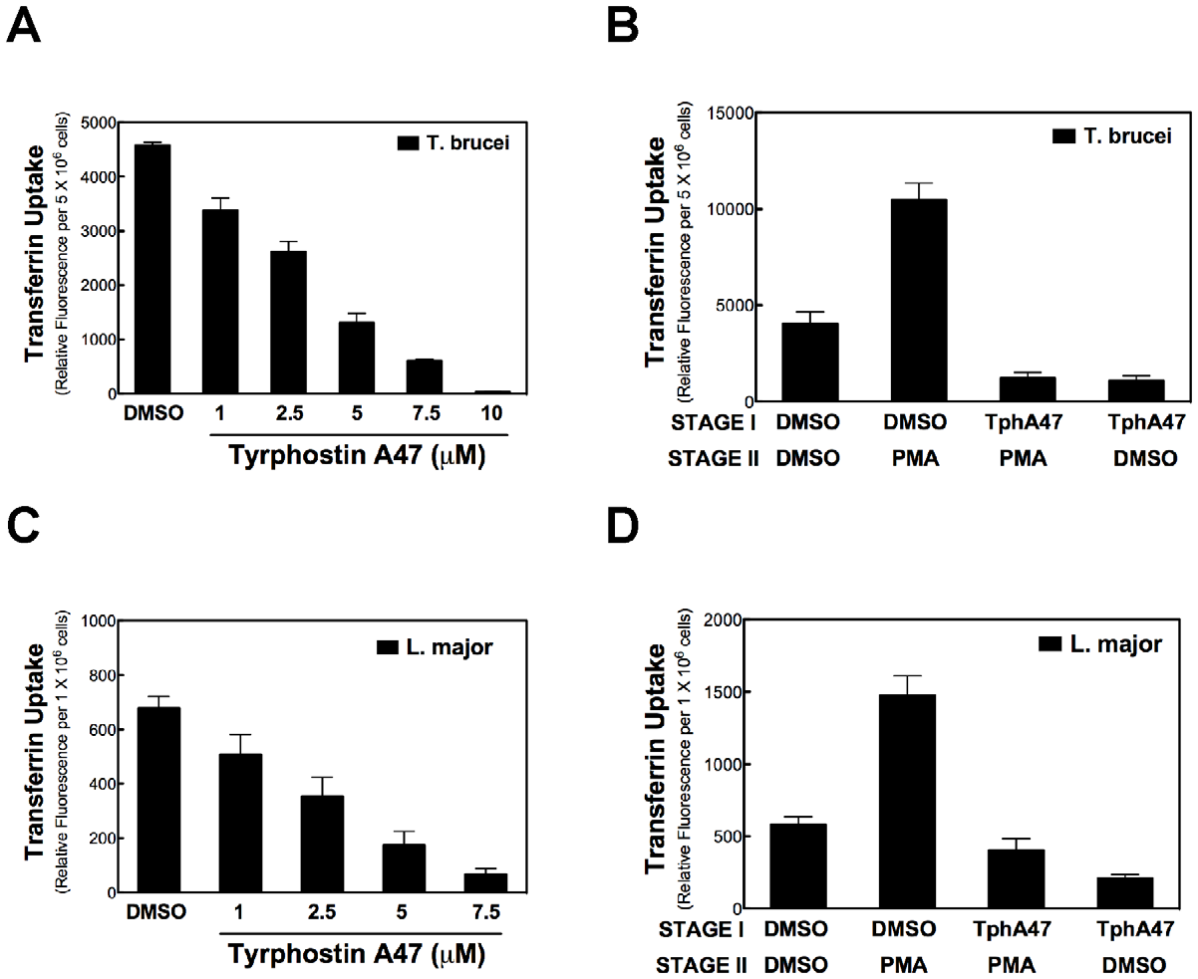
A PTK inhibitor blocks phorbol ester-stimulated endocytosis of Tf in *T. brucei* and *L. major*. *(* ***A*** *) T. brucei* (5×10^6^) were incubated with varying concentrations of Tyrphostin A47 for 10 min. Parasites were rinsed, and endocytosis of Tf measured (see legend to Fig. 1 for protocol). *(* ***B*** *) T. brucei* (5×10^6^ cells) was incubated in medium containing Tyrphostin A47 (TphA47) (7.5 µM) for 10 min (37°C) (i.e. Stage I). Cells were then exposed to PMA (500 nM) (Stage II) for another 10 min, and endocytosis of Tf was measured. *(* ***C*** *) L. major* (5×10^6^) were incubated with varying concentrations of Tyrphostin A47 for 10 min. Parasites were rinsed, and endocytosis of Tf measured (see legend to Fig. 1 for protocol). *(* ***D*** *) L major* (5×10^6^/ml) was treated with Tyrphostin A47 (5 µM) for 15 min in culture medium. Thereafter, cells were incubated with PMA (500 nM; final conc.) for 15 min, and endocytosis of Tf was measured as described the legend to Figure 1.

Preincubation of *T. brucei* with TphA47 diminished Tf uptake greater than 90% (Fig. 7A). We infer that the pathway for endocytosis of Tf in *T. brucei* involves activity of a TbPTK. Next, we determined whether PMA-activated Tf endocytosis required a TbPTK. *T. brucei* were pre-incubated with TphA47 before PMA was added, and Tf endocytosis quantitated. When TphA47 was added to cells prior to introduction of PMA, stimulation of Tf endocytosis by PMA was blocked (Fig. 7B). From these data we conclude that a TbPTK is required for PMA-activated endocytosis of Tf in *T. brucei*. In sharp contrast, a Ser/Thr kinase (*e.g.* PKC) is not needed in the pathway for PMA-stimulated endocytosis of Tf in the trypanosome (Fig. 5B). Similar studies were performed with *L. major*, since PMA and DAGs activated endocytosis of Tf in the parasite (Fig. 4A and Fig. 4B). TphA47 inhibited Tf endocytosis in *L. major* (IC_50_ of 2.5 µM) (Fig. 7C). Preincubation of *L. major* with TphA47 before addition of PMA led to a 400% reduction of phorbol ester-activated uptake of Tf, as compared to cell that were treated with PMA in absence of TphA47 (Fig. 7D). Thus, PTKs in *L. major* act downstream of PMA activation of Tf endocytosis in the parasite, similar to our observations with *T. brucei* (Fig. 7B).

## Discussion

### Transferrin Endocytosis in Trypanosomatids Is Stimulated by Diacylglycerol

Iron is important for viability of *T. brucei* and *Leishmania* species [13], [37], [38], [39], [40]. In both trypanosomatids the metal ion is acquired by endocytosis, following its binding to Tf which has a receptor (or binding protein) at the plasma membrane [14], [41], [42]. Some major components of the endocytic machinery (e.g., clathrin, Rabs and SNAREs) affect endocytosis of Tf in trypanosomatids. However, small molecule and/or lipid regulators of Tf endocytosis have not been described. Thus, our demonstration that DAG regulates Tf endocytosis in both *T. brucei* and *L. major* presents an opportunity to study the mechanisms by which the lipid influences the endocytic system in these deeply-diverged eukaryotes.

In *L. major*, stable expression of a GPI-PLC activates endocytosis of Tf (Fig. 1A and Fig. 3). Interestingly, the intracellular location of the enzyme either on endosomes (*e.g.*, unmutated GPI-PLC) or glycosomes (*e.g.*, Cys269,270,273Ser mutant of GPI-PLC) [43] does not change the effect of the enzyme on Tf endocytosis (Fig. 2). These data are consistent with a proposal that GPI-PLC releases a diffusible product (diacylglycerol (DAG)) that mediates the effect of the enzyme on Tf endocytosis. In support of this claim, exogenous DAG (or PMA, a DAG mimic) stimulate uptake of Tf as predicted (Fig. 4).

### Tyrosine Kinases Are Effectors for DAG-Regulated Endocytosis in *T. brucei and L. major*

**Figure 8:**
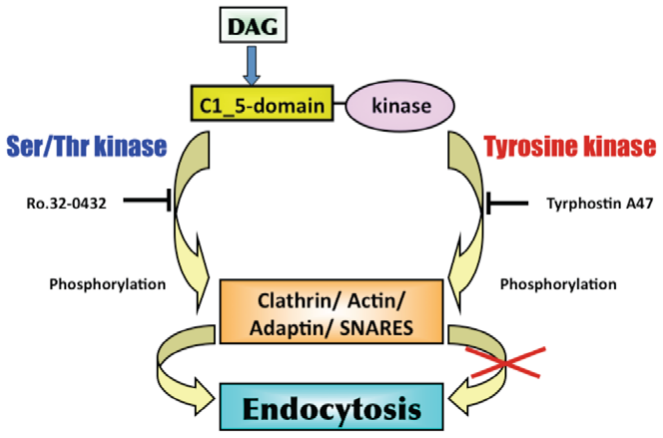
A Working Model for DAG Activation of Tf Endocytosis in Trypanosomatids. Based on our biochemical (Figs. 1 through Fig. 6), bioinformatic (Fig. 6, Table 1), and pharmacological data (Fig. 7), we propose that DAG binds to a C1_5 domain of a PTK in *T. brucei* (or *L. major*). The trypanosomatid PTK is activated by DAG, and the enzyme phosphorylates components of the endosomal pathway to activate uptake of Tf.

Our data indicate that a PTK is involved in DAG signaling for endocytosis of Tf in *T. brucei* (Fig. 7B) and *L. major* (Fig. 7C). Participation of Tyr kinase, instead of a Ser/Thr kinase (Fig. 5B), in DAG signaling is supported by bioinformatic analysis of the parasite genome. Whereas the protein Tb11.01.2290 (Table 1) contains both PTK and C1_5 domains, no Ser/Thr kinase in the genomes of either *Leishmania* or *T. brucei* has a C1_5 domain. Therefore neither *T. brucei* nor *Leishmania* has a classic PKC. We speculate that Tb11.01.2290 in *T. brucei* and LmjF36.5350 in *L. major* could be effector PTKs for DAG regulation of Tf endocytosis in the parasites. The enzymes may activate endocytosis by phosphorylating clathrin, actin, adaptins or other core components of the endocytosis machinery. In vertebrates, the cytoplasmic PTKs Src, Abl and Lyn modulate endocytosis by phosphorylating components of the endocytic machinery (e.g. clathrin, AP2, dynamin and WASP) (reviewed in [44]). Our working hypothesis for the role of PTKs and C1_5-domain proteins in DAG-regulated endocytosis in *T. brucei* or *L. major* is summarized in Fig. 8.

### Diacylglycerol Signaling Pathways in Trypanosomatids and Vertebrates

DAG is an intracellular second messenger for signaling in eukaryotes. In vertebrates, ligands for receptor tyrosine kinases and seven transmembrane receptors can activate phosphatidylinositol phospholipases C that cleave phosphoinositides. DAG released by the phospholipases C activates PKC to phosphorylate several substrates. Due to the multiplicity of its targets, PKC can regulate several pathways including apoptosis, cell proliferation, differentiation, and endocytosis (reviewed in [45]). Receptor endocytosis in vertebrates can be controlled by phorbol esters without PKC participation. For example, Munc13 which lacks a kinase domain binds DAG and regulates exocytosis by interacting with Rab and SNARE [46], [47].

Trypanosomatid genomes do not encode the most common eukaryote signaling systems (*e.g.* receptor Tyr kinases, heterotrimeric G proteins, and G-protein coupled receptors). Consequently few signaling pathways have been characterized completely in trypanosomatids (reviewed in [48], [49]). Signaling with intracellular second messengers has four general components, namely, (i) ligands, (ii) receptors, (iii) effectors, and (iv) physiological targets. DAG is a signaling ligand in trypanosomatids (Fig. 4, Fig. 5 and Fig. 7) and in vertebrates ([2]. However, apart from the ligand it appears that other components in the pathway have diverged from those in humans.

First, DAG receptors in vertebrates contain a classic C1-domain [50]. Surprisingly, the vertebrate C1-domain is absent from the genomes of *L. major* and *T. brucei*. Instead we found a divergent C1_5 domain that might be the receptor for DAG in trypanosomatids (Fig. 6). Second, in vertebrates a majority of DAG signaling pathways depend on Ser/Thr kinases (PKCs) [26], [51], [52]. In contrast, DAG-stimulated endocytosis of Tf in *T. brucei* and *L. major* depends on protein Tyr kinase (Fig. 7B and Fig. 7D). Thus, effector enzymes for DAG signaling in *L. major* and *T. brucei* are Tyr kinases instead of Ser/Thr kinases. Finally, enhanced uptake of Tf in response to DAG (or phorbol ester) treatment is unique to trypanosomatids. In human cells, PMA treatment does not increase endocytosis of Tf [53], [54]. During a trypanosome infection of vertebrates, the parasite competes with host cells for Tf in host blood. Therefore, DAG stimulation of Tf uptake may contribute to virulence of the parasite by helping *T. brucei* to acquire sufficient amounts of the ligand (and indirectly iron) to sustain its extracellular existence in host blood.

In brief, *L. major*, *T. brucei* and vertebrates use DAG as a second messenger for intracellular signaling. However, the effector enzymes are not identical, and by extension the targets of the activated kinases could be different. Consequently, studies of DAG signaling pathways in trypanosomes are likely to yield new insights into the diversity of DAG signaling in eukaryotes.

## Materials and Methods

### Cell Culture

Transfected *Leishmania major* CC1 expressing *GPI-PLC* (pUTK-GPIPLC) and vector alone (pUTK) [17] were grown at 27°C to a maximum density of 5×10^6^/ml in M199 (supplemented with 5% FBS, 40 mM HEPES, 0.0005% hemin, 0.0001% biotin, 0.1 mM adenine, and 1% antibiotic-antimycotic) [55] in presence of 50 µg/ml G418. *T. brucei* RUMP 528 (from Dr. George Cross (Rockefeller University) was cultured as described [7]. All *Leishmania* studies were performed with promastigote (insect stage) in logarithmic phase of growth.

### Materials

Restriction enzymes were from New England Biolabs (Beverly, MA); Nonidet P40 (NP40) was from Calbiochem (San Diego, CA). Fetal bovine serum and newborn calf serum were from Hyclone (Logan, UT). G418 was from Fisher Scientific (Norcross, GA). Transferrin- Alexa Fluor-594 was purchased from Molecular Probes (Eugene, OR). Phorbol-12-myristate-13-acetate and 4α-phorbol-12-myristate-13-acetate were from Sigma (St. Louis, MO). Hygromycin, phleomycin, Tyrphostin A47, and Ro.32-0432 were obtained from Calbiochem (USA). All other reagents were from Sigma (St. Louis, MO).

### DNA Transfection of *Leishmania major*

*Leishmania major* was cultured at 27°C to a density of 10^7^/ml in M199 [56], and transfected as described [43]. Twelve hours post-transfection, G418 (dissolved in PBS and filter sterilized) was added to a final concentration of 30 µg/ml. Stable transfectants were maintained in M199 medium containing 50 µg/ml G418.

### Transferrin Endocytosis Assays

*Leishmania* was cultured to a density of 5×10^6^ ml^−1^. Cells (1×10^8^) were pelleted, rinsed with buffer containing 50 mM bicine, 50 mM NaCl, 5 mM KCl, 1% glucose, pH 7.4 (BBS/G), and harvested (1,400×g for 5 min at room temperature). The cell pellet was resuspended in cold serum-free IMDM (Iscove's modified Dulbecco's medium), and stored on ice for 10 min. Transferrin–Alexa Fluor® 594 ((Molecular Probes, OR) was added at 25 µg/ml (final concentration) and cell suspension were incubated at 27°C. At indicated time intervals, aliquots of cells (1×10^6^) were withdrawn and pelleted at 5000 g for 5 min at 4°C. The cell pellet was washed five times with ice-cold BBS/G containing 2% (w/v) sodium azide (5000×g for 3 min at 4°C), resuspended in 100 µl of the same buffer (ice-cold) and deposited into 96-well plates that were kept on ice at all times. For *T. brucei*, endocytosis of transferrin-Alexa Fluor 594 was performed as described earlier ([7]). Values obtained for a “blank” (*i.e.* without Tf) was subtracted from experimental readings, and relative fluorescence units were plotted. Each time point is a mean (±std deviation) of triplicate measurements.

Phorbol ester and diacylglycerols were pre-incubated with *L. major* at 27°C (or *T. brucei* at 37°C) for 15 min at concentrations stated in the respective figures. Cells were washed with ice-cold serum-free IMDM medium before transferrin uptake was documented as described above.
